# Comparison of bacterial and archaeal communities in two fertilizer doses and soil compartments under continuous cultivation system of garlic

**DOI:** 10.1371/journal.pone.0250571

**Published:** 2021-05-14

**Authors:** Jing Zhou, Yong Kong, Wangfeng Zhao, Guangshan Wei, Qingfeng Wang, Longchuan Ma, Taotao Wang, Fengyue Shu, Weilai Sha

**Affiliations:** 1 School of Life Sciences, Qufu Normal University, Jining, PR China; 2 College of Biological and Environmental Engineering, Binzhou University, Binzhou, PR China; 3 South China Sea Resource Exploitation and Protection Collaborative Innovation Center (SCS-REPIC) / School of Marine Sciences, Sun Yat-Sen University, Guangzhou, China; 4 Key Laboratory of Marine Genetic Resources, Ministry of Natural Resources of the PR China, Third Institute of Oceanography, Xiamen, China; 5 Eco-environmental Protection Research Institute, Shanghai Academy of Agricultural Sciences, Shanghai, PR China; 6 Shandong Engineering and Technology Research Center for Garlic, Jining, PR China; Graduate University of Advanced Technology, Kerman Iran, ISLAMIC REPUBLIC OF IRAN

## Abstract

Soil microbial communities are affected by interactions between agricultural management (e.g., fertilizer) and soil compartment, but few studies have considered combinations of these factors. We compared the microbial abundance, diversity and community structure in two fertilizer dose (high vs. low NPK) and soil compartment (rhizosphere vs. bulk soils) under 6-year fertilization regimes in a continuous garlic cropping system in China. The soil contents of NO_3_^–^ and available K were significantly higher in bulk soil in the high-NPK. The 16S rRNA gene-based bacterial and archaeal abundances were positively affected by both the fertilizer dose and soil compartment, and were higher in the high-NPK fertilization and rhizosphere samples. High-NPK fertilization increased the Shannon index and decreased bacterial and archaeal richness, whereas the evenness was marginally positively affected by both the fertilizer dose and soil compartment. Soil compartment exerted a greater effect on the bacterial and archaeal community structure than did the fertilization dose, as demonstrated by both the nonmetric multidimensional scaling and redundancy analysis results. We found that rhizosphere effects significantly distinguished 12 dominant classes of bacterial and archaeal communities, whereas the fertilizer dose significantly identified four dominant classes. In particular, a Linear Effect Size analysis showed that some taxa, including Alphaproteobacteria, Rhizobiales, Xanthomonadaceae and *Flavobacterium*, were enriched in the garlic rhizosphere of the high-NPK fertilizer samples. Overall, the fertilizer dose interacted with soil compartment to shape the bacterial and archaeal community composition, abundance, and biodiversity in the garlic rhizosphere. These results provide an important basis for further understanding adaptive garlic-microbe feedback, reframing roots as a significant moderating influence in agricultural management and shaping the microbial community.

## Introduction

Many studies have confirmed that the rhizosphere microbiota is a subset of the entire bulk soil community [1]. However, the continuous cropping of Dihuang (*Rehmannia glutinosa*) [2], strawberries (*Fragaria ananassa*) [3], and maize (*Zea mays L*.) [4] has different effects on the bacterial communities in different soil compartment (bulk and rhizosphere) due to the additional complexity resulting from roots. Furthermore, changes in nutrient availability resulting from fertilization regimes can affect the rhizosphere microbiome by altering the root morphology and root exudation [5]. Previous studies have focused on the effects of fertilization regimes on various microbial groups in soil, including bacteria [6], fungi [7] and functional microbiota (e.g., ammonia-oxidizing archaeal communities) [8]. However, the combined influence of fertilization regime and rhizosphere effects on bacterial communities is not well understood.

Garlic (*Allium sativum* L), which is used worldwide as a flavouring agent and cover crop, is an important vegetable and medicinal plant [9]. China ranks first worldwide in terms of cultivated area (7,914,000 hectares) and output (2,005.84 million tonnes) of garlic (Food and Agriculture Organization, 2017). Garlic root exudates transfer allelochemicals to the soil environment [10] and selectively re-establish microbial communities in the rhizosphere, which results in the creation of taxonomically distinct microbial communities from bulk soil [11]. Relevant data have shown that the number of beneficial microorganisms and the level of enzyme activity in the soil initially increase and then decrease with continuous garlic cropping years [12]. After 15–20 years of continuous garlic cropping, various factors in the soil system are disordered, resulting in an imbalance in the soil microbial population structure [12]. In addition to an increase in soil fungi, other positive indicators of the growth and development of garlic, such as soil microbial species and soil ammoniated bacteria, show downward trends, which results in serious obstacles to continuous cropping and annual reductions in the garlic yield [13]. It is necessary to explore how fertilization regimes and plant selection affect rhizosphere bacterial and archaeal communities and to consider how they interact in combination rather than individually. However, few studies have focused on the effects of the rhizosphere and fertilizer regimes on bacterial and archaeal communities in continuous garlic cropping systems.

The aims of this study were to (i) compare the changes in the compositions of bacterial and archaeal communities under different doses of chemical fertilizer; (ii) compare the changes in the bacterial and archaeal community compositions of the bulk soil and garlic rhizosphere; and (iii) determine the main factors shaping the bacterial and archaeal community structure.

## Methods

### Ethics statement

This study was carried out on private land and the owner of the land gave permission to conduct the study on this site. The study was observational, involving no cruelty to animals, no damage to habitats and no harm to endangered plants, and thus no review from the ethnic committee was required in China. All the work was carried out under the Wildlife Protection Law of the People’s Republic of China.

### Sample collection

The site of the garlic test field was in Gaohe town, Jinxiang County, Shandong Province, China (35°05′11″N, 116°22′38″E) and was established in 2010. The soil is a typical calcareous fluvo-aquic soil (aquic inceptisol) of the North China Plain, consisting of a calcareous, fluvo-aquic sandy loam [14]. The initial soil nutrient status before the experiment was analysed at the Shandong Engineering and Technology Research Center for Garlic (Jining, Shandong Province, China), and the results are shown in Table 1. One garlic cultivar, ‘Caijiapo Red Skin’ (G026), was grown in the field starting in early September 2015 and ended in late July 2016. Considering it is impossible to grow garlic locally in the region without applying fertilizer, five experimental replicates were conducted using two fertilization treatments in a randomized complete block design. There is a recommended dose of Nitrogen (N), phosphorus (P) and Potassium (K)fertilizers of garlic for towns in Jinxiang County (such as Huayu, Yushan and Mamiao): N_2_ = 450 kg/ha, P_2_O_5_ = 240 kg/ha, K_2_O = 360 kg/ha [15]. However, there is no recommended dose in Gaohe town, and High and Low doses were selected according to the amount used by local farmers. The 36-m^2^ (9 m × 4 m) test field received two fertilizer treatments refer to the local fertilization amount of NPK fertilizer: a low dose of NPK fertilizer (kg/ha) [135 N (urea), 76.5 P_2_O_5_ (Calcium phosphate) and 135 K_2_O (potassium sulphate)] and a high dose of NPK fertilizer (kg/ha) (270 N, 135 P_2_O_5_, and 270 K_2_O). Full does of calcium phosphate (to meet the phosphorus needs of garlic cloves during germination) andcalcium sulphate (to help garlic have a well-developed root system, strong stems and control against pythium root rot and soft rot [16]), 1/3 urea and potassium sulphate were applied as the basal fertilizer five days before planting, and the remaining 2/3 of urea and potassium sulphate were applied as a top dressing during the garlic bolting period. All other garlic management measures followed local practices.

**Table 1:** Initial soil nutrient status before the experiment.

pH	Organic Matter (g/kg)	Total N (g/kg)	Avail K (mg/kg)	Avail P (mg/kg)	NH_4_^+^ -N
					(mg/kg)
8.24	5.7	2.47	18	61.0	151.l

In May 2016 (at the garlic bolting stage), six garlic root samples in each plot were randomly collected and pooled together as the rhizosphere soil sample, according to Mcpherson et al. [17]. The bulk soil was collected according to Zhou et al. [6]. The bulk soil samples from the high- and low-NPK treatments were denoted HO and LO, respectively, while the corresponding rhizosphere soil samples were named HR and LR. All 20 samples were placed into individually-labelled zipper storage bag and put in cooling container with ice (2–8°C) and transported to the laboratory. All soil samples were sieved (2 mm) to remove garlic roots and were evenly divided into two subsamples; one of the subsamples was air-dried, and the other was stored at −80°C for DNA extraction.

### Soil chemical properties and garlic yield

The soil pH was determined by a pH electrode at a soil-to-water ratio of 1:2.5 [18]. The organic matter (OM) was determined from weight loss according to Parker [19], and the total nitrogen (total N) was determined according to Sims [18]. The soil KCl extractable nitrate (NO_3_^−^) and ammonium (NH_4_^+^) concentrations were determined according to Sparks et al. [20]. The soil available phosphorus (Avail P) was determined at a soil-to-sodium bicarbonate ratio of a 1:10 by Olsen’s procedure, and the available potassium (Avail K) was analysed after extraction at a soil-to-ammonium acetate ratio of 1:10 by an ion-exchange resin procedure [21]. The garlic yield was determined by weighing the collected bulbs, and this value was used to estimate the yield per hectare [22].

### High-throughput sequencing analysis

The total genomic DNA from the soil samples was extracted using the PowerSoil DNA Extraction Kit (MO BIO Laboratories, Carlsbad, CA) according to the manufacturer’s recommended protocol [23]. The DNA was amplified with the primers 515F and 806R [24]. The reverse primer contained a unique 6-bp error-correction barcode for each sample. After library construction of the 16S rRNA gene fragments, high-throughput sequencing was performed on the Illumina MiSeq 2 x 250 platform at Shanghai Majorbio Bio-pharm Technology Co., Ltd., China. The original sequences are stored in the NCBI sequence read archive (accession number PRJNA531809).

Paired end reads were processed using the Quantitative Insights into Microbial Ecology (QIIME) software pipeline according to standard protocols [25]. Presumptive chimeric sequences were screened and discarded using UCHIME [26]. Relevant non-chimeric sequence groups were assigned to operational taxonomic units (OTUs) with a maximum classification distance of 3%. The α-diversity in each sample was calculated using the UPARSE pipeline [27]. The representative sequences of each subsampled OTU were classified and assigned using SINA aligner (version 1.1) [28] and the SILVA 16S rRNA database [29].

### qPCR analysis

The abundance of bacterial and archaeal 16S rRNA was determined by ABI 7500 real-time PCR according to Zhou et al. [6]. Briefly, the PCR conditions were as follows: the enzyme was activated for 1 min at 95°C followed by 40 cycles of 15 s at 94°C, 34 s at 55°C and 15 s at 72°C. All PCR assays were performed using Maxima® SYBR Green/ROX qPCR Master Mix (Qiagen, Germantown, MD, USA), as specified by the manufacturer. Interesting gene fragments were cloned from the amplified 16S rRNA gene using the 515f/806r primers. The linearized plasmid was diluted 10 times to produce a standard template. The standard curve exhibited an R^2^ > 0.99.

### Statistical analysis

We used the Kolmogorov-Smirnov test to determine whether the sample data were normally distributed before other analyses. The differences in the chemical properties, α-diversity indices and relative abundances of the dominant groups were tested using a one-way analysis of variance by using the "aov" function in R v. 3.6.1. Pearson correlation coefficients between the soil properties and bacterial and archaeal diversity and abundance were calculated using SPSS 19.1. In all trials, *P* < 0.05 was considered significant. The bacterial and archaeal genera that were differentially represented between the four soil sample types were measured by coupling the linear discriminant analysis (LDA) and with the effect size (LEfSe) [30, 31]. For analysis of the β-diversity, a nonmetric multidimensional scaling (NMDS) ranking of Hellinger distances was performed using the cmdscale function [32]. Permutational multivariate (999) analysis of variance (PERMANOVA) was performed to assess the differences in the pairwise combinations (HO-HR, HO-LO, HO-LR, HR-LO, HR-LR and LO-LR) to complement the ANOSIM results at the genus level. A redundancy analysis (RDA) was performed to explore the possible linkages between bacterial and archaeal communities at the genus level and soil properties using CANOCO 5.0 and a Monte Carlo test with 499 permutations.

## Results

### Soil chemical properties

The soil pH value ranged from 7.65 to 8.08 among the four experimental groups (Table 2), and there was no significant difference in soil pH among the experimental groups. However, the soil pH was lower at the end of the experiment (Tables 1 and 2); this phenomenon may be caused by the long-term accumulation of organic acids (i.e. acetic acid and maleic acid) and acidic amino acids (i.e. aspartic acid) in garlic root exudates [33], and this effect is greater than the alkalinity brought by calcium addition. Similar results were observed for the soil contents of Avail P and organic matter (Tables 1 and 2). Soil contents of NO_3_^–^, Avail K and total N were significantly higher in HO and HR than in LO and LR, respectively (Table 2). Furthermore, higher levels of NO_3_^–^ and Avail K but lower NH_4_^+^ were observed in HO compared to HR, which could be caused garlic roots having a strong ability to absorb NO_3_^–^, instead of NH_4_^+^ [34]. No significant difference in soil properties was detected between LO and LR (Table 2). However, the six years of high-NPK fertilizer application significantly increased the garlic yield from 48.2% to 64.0% compared with the yield obtained with the low-NPK fertilizer samples during 2011–2016 (S1 Fig).

**Table 2:** Soil chemical properties under different soil groups.

Group	pH	Avail K	Total N(mg/kg)	Avail P	NH_4_^+^	NO_3_^–^	OM
		(mg/kg)		(mg/kg)	(mg/kg)	(mg/kg)	(g/kg)
HO	8.00±0.01a	6.25±0.06d	1.62±0.06b	56.72±3.25a	15.24±0.23a	15.25±0.1c	1.73±0.09a
HR	8.05±0.01a	6.08±0.02c	1.69±0.01b	52.84±0.22a	19.3±1.22b	9.49±0.57b	1.72±0.06a
LO	8.08±0.04a	3.76±0.05a	1.44±0.03a	56.08±1.35a	16.72±0.24a	8.21±0.21a	1.56±0.11a
LR	7.65±0.49a	3.61±0.03a	1.47±0.03a	56.72±2.15a	16.12±2.37a	7.81±0.13a	1.58±0.08a

Values are mean± standard deviation (*N* = 3). Values within the same column followed by different letters indicate significant difference (*P*<0.05).

### Bacterial and archaeal 16S rRNA gene copy number

**Fig 1:**
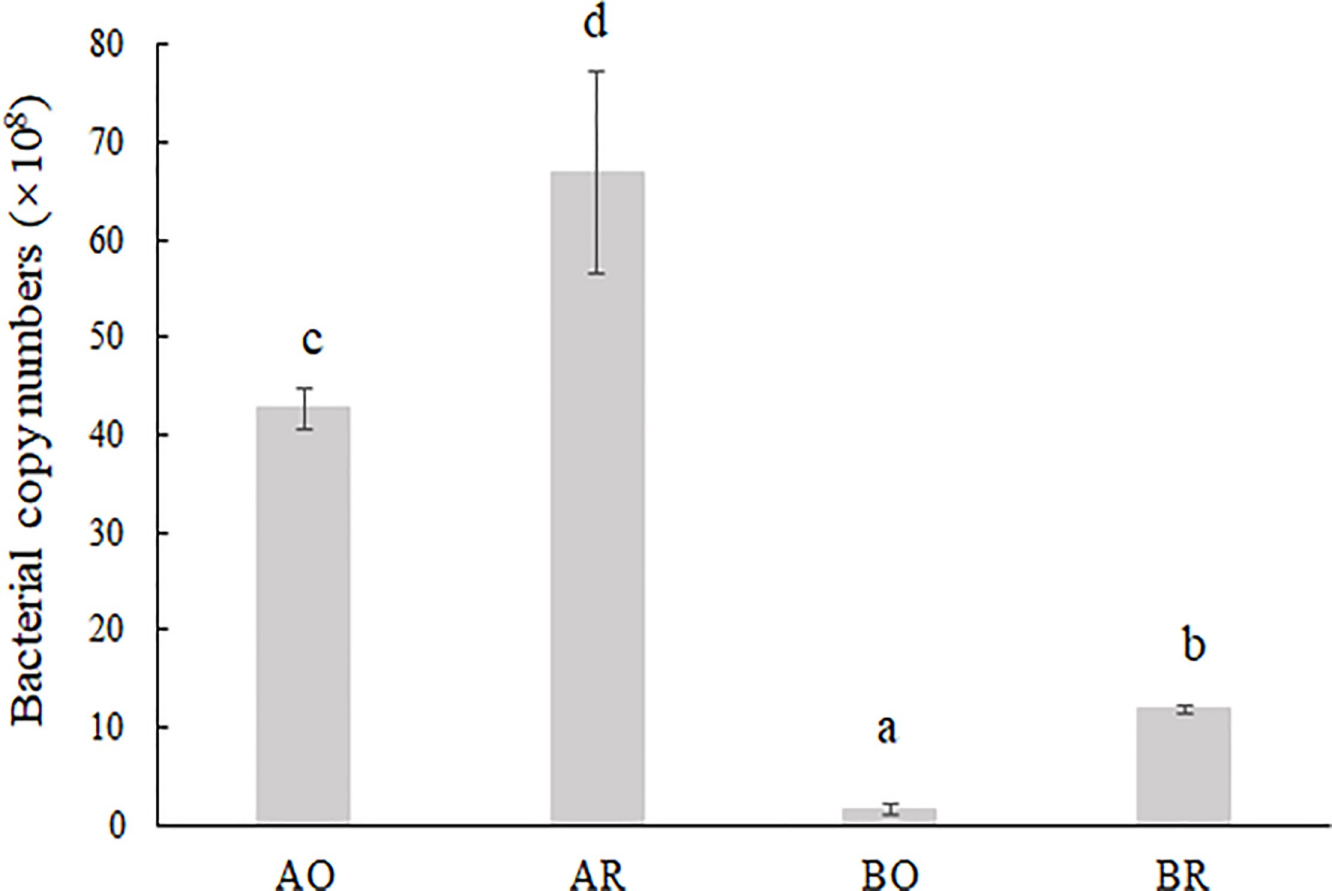
Abundances of bacteria, as indicated by the number of 16S rDNA copies measured by quantitative PCR.

We observed the 16S rRNA copy number in four treatments ranged from 1.5 × 10^8^ to 4.3 × 10^9^ copies in 1 g of wet soil (Fig 1). The bacterial and archaeal abundances in the rhizosphere were significantly higher than those in the bulk soil in both the high- (HR: HO = 1.57) and low- (LR:LO = 7.90) NPK samples. Furthermore, higher 16S rRNA copy numbers were observed in the high-NPK treatment than in the low-NPK treatment in both the rhizosphere (HR:LR = 5.65) and bulk (HO:LO = 28.43) soils (Fig 1). The bacterial and archaeal abundances were positively correlated with the concentrations of Avail K (*r* = 0.895, *P* < 0.01), total N (*F* = 0.955, *P* < 0.01) and organic matter (*r* = 0.664, *P* < 0.05) (S1 Table).

### Bacterial and archaeal α-diversity analysis

After the selection and chimaera analysis of the OTUs, 1,296,155 high-quality sequences were assigned to 6,484 non-singleton OTUs, resulting in the classification of 612 taxa at the genus level. Based on a similarity cutoff of 97%, Good’s coverages ranged from 96.5% to 99.0% (Table 3), which indicated that the number of reads were sufficient to represent the bacterial and archaeal diversity in all the samples.

**Table 3:** Bacterial and and archaeal alpha diversity in four soil samples.

Group	Coverage	ACE	Chao	Shannon	Shannoneven
HO	0.97±0.028a	5591±353a	5186±672a	6.22±0.09b	0.779±0.023ab
HR	0.97±0.009a	5323±382a	5088±435a	6.23±0.12b	0.784±0.017b
LO	0.98±0.005a	5898±198a	5718±167b	6.09±0.07a	0.756±0.013a
LR	0.98±0.013a	5592±503a	5429±351b	6.04±0.06a	0.779±0.016ab
*P-*value	0.34	0.16	0.031	0. 033	0.08

Values are mean± standard deviation (N = 5). Values within the same column followed by different letters indicate significant difference (*P*<0.05).

No obvious differences (*P* > 0.05) in ACE were found among the four soil samples. However, the Shannon and Chao indices in low NPK treatment were significantly different, compared to high NPK fertilizer soils. The Shannon index of both the rhizosphere and bulk samples was significantly lower under the low-NPK treatment compared to the high-NPK treatment, whereas the Chao index showed the opposite trend (*P* < 0.05, Table 3). The evenness (Shannoneven) was marginally positively affected by both soil compartment and fertilizer dose. Compared with the Shannoneven in the bulk soil (HO and LO), the values in the respective rhizosphere samples (HR and LR) were slightly higher (Table 3). Similarly, the Shannoneven values obtained for the high-NPK fertilizer samples (HO and HR) were slightly higher than those found for their respective low-NPK fertilizer samples (LO and LR) (Table 3).

The Shannon index was positively and significantly correlated with the soil NH_4_^+^ content, and ACE was negatively and significantly (*F* = 0.447, *P* < 0.05, S2 Table) correlated with the total N. However, no obvious correlation was found between the Chao index and the measured soil properties (S2 Table).

### Bacterial and archaeal composition

**Fig 2:**
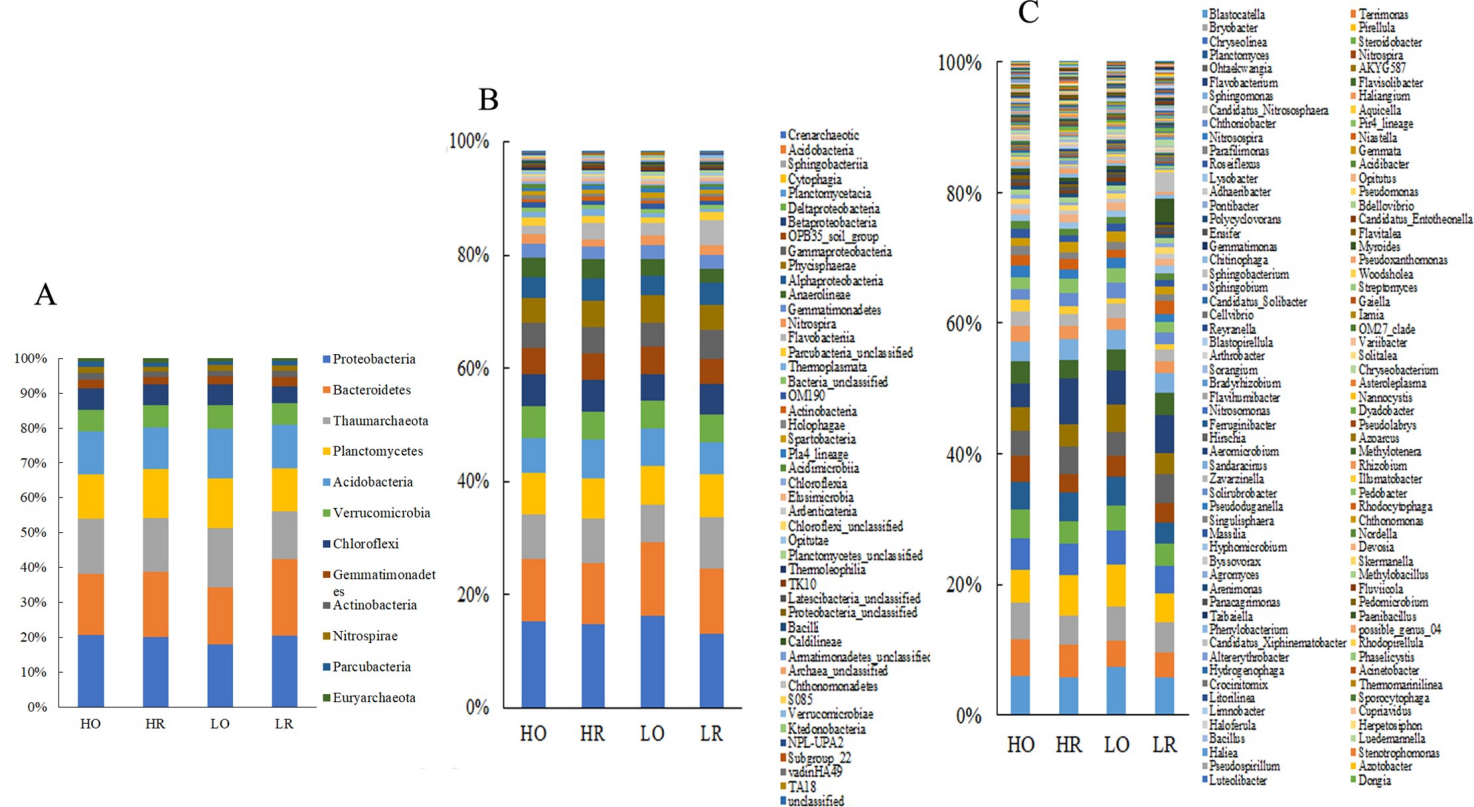
Relative average abundances of the most abundant taxa across soil groups. A. Phyla (relative abundance >1%), B. Classes (relative abundance >0.1%), C. Genera (relative abundance >0.01%).

Proteobacteria, Bacteroidetes, Thaumarchaeota, Planctomycetes and Acidobacteria were the top five dominant phyla and accounted for 76.4–78.2% of the bacterial sequences obtained from the soil samples (Fig 2A). The top dominant classes were the Soil_Crenarchaeotic_Group (13.2–16.3%), Acidobacteria (10.8–13.0%), Sphingobacteriia (6.9–7.8%) and Cytophagia (6.9–7.8%) (Fig 2B). The top five most abundant genera were Blastocatella (1.9–2.4%), Terrimonas (1.3–19%), Bryobacter (1.5–1.8%), Pirellula (1.7–2.2%) and Chryseolinea (1.6–1.7%) (Fig 2C).

**Fig 3:**
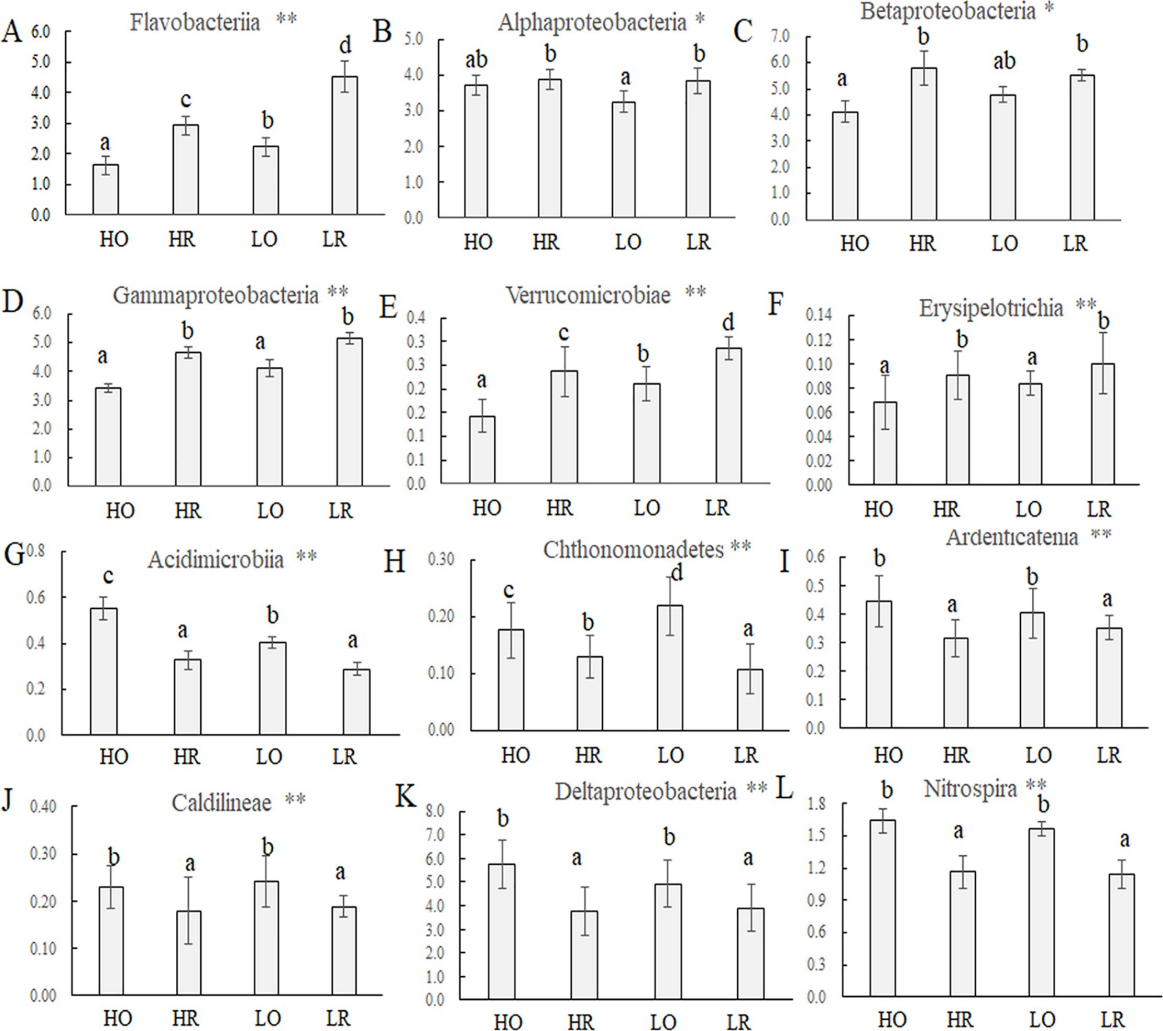
Average relative abundances (%) of classes affected by rhizosphere effects. The error bars indicate the standard deviations of the relative abundances between five replicate samples.

Soil compartment significantly distinguished 12 classes of bacteria and archaea, as shown in Fig 3. The classes Flavobacteriia, Alphaproteobacteria, Betaproteobacteria, Gammaproteobacteria, Verrucomicrobiae and Erysipelotrichia were significantly enriched (*P* < 0.05) in the rhizosphere relative to bulk soil (Fig 3A–3F and Table 4), whereas the classes Acidimicrobiia, Chthonomonadetes, Ardenticatenia, Caldilineae, Deltaproteobacteria and Nitrospira were more abundant (*P*<0.05) in the bulk soil than in the rhizosphere (Fig 3G–3L, Table 4).

**Table 4:** Relative abundance (%) of classes under different fertilizer treatments.

Classes	HO	HR	LO	LR	Thermal classifications
Flavobacteriia	1.62	2.92	2.21	4.53	Mesophilic
Alphaproteobacteria	3.72	3.88	3.25	3.84	Psychrophilic
Betaproteobacteria	4.13	5.80	4.79	5.51	Psychrophilic
Gammaproteobacteria	3.42	4.65	4.10	5.14	Psychrophilic
Verrucomicrobiae	0.14	0.24	0.21	0.29	Mesophilic
Erysipelotrichia	0.07	0.09	0.08	0.10	Mesophilic
Acidimicrobiia	0.55	0.33	0.40	0.29	Thermophilic
Chthonomonadetes	0.18	0.13	0.22	0.11	Thermophilic
Ardenticatenia	0.45	0.41	0.40	0.35	Thermophilic
Caldilineae	0.23	0.18	0.24	0.19	Thermophilic
Nitrospira	1.64	1.16	1.57	1.14	Thermophilic
Deltaproteobacteria	5.75	3.76	4.92	3.90	Psychrophilic
Anaerolineae	3.44	3.50	3.13	2.52	Thermophilic
Ktedonobacteria	0.14	0.13	0.17	0.18	Thermophilic
Fibrobacteria	0.04	0.04	0.05	0.06	Mesophilic
Thermomicrobia	0.01	0.00	0.01	0.00	Thermophilic

**Fig 4:**
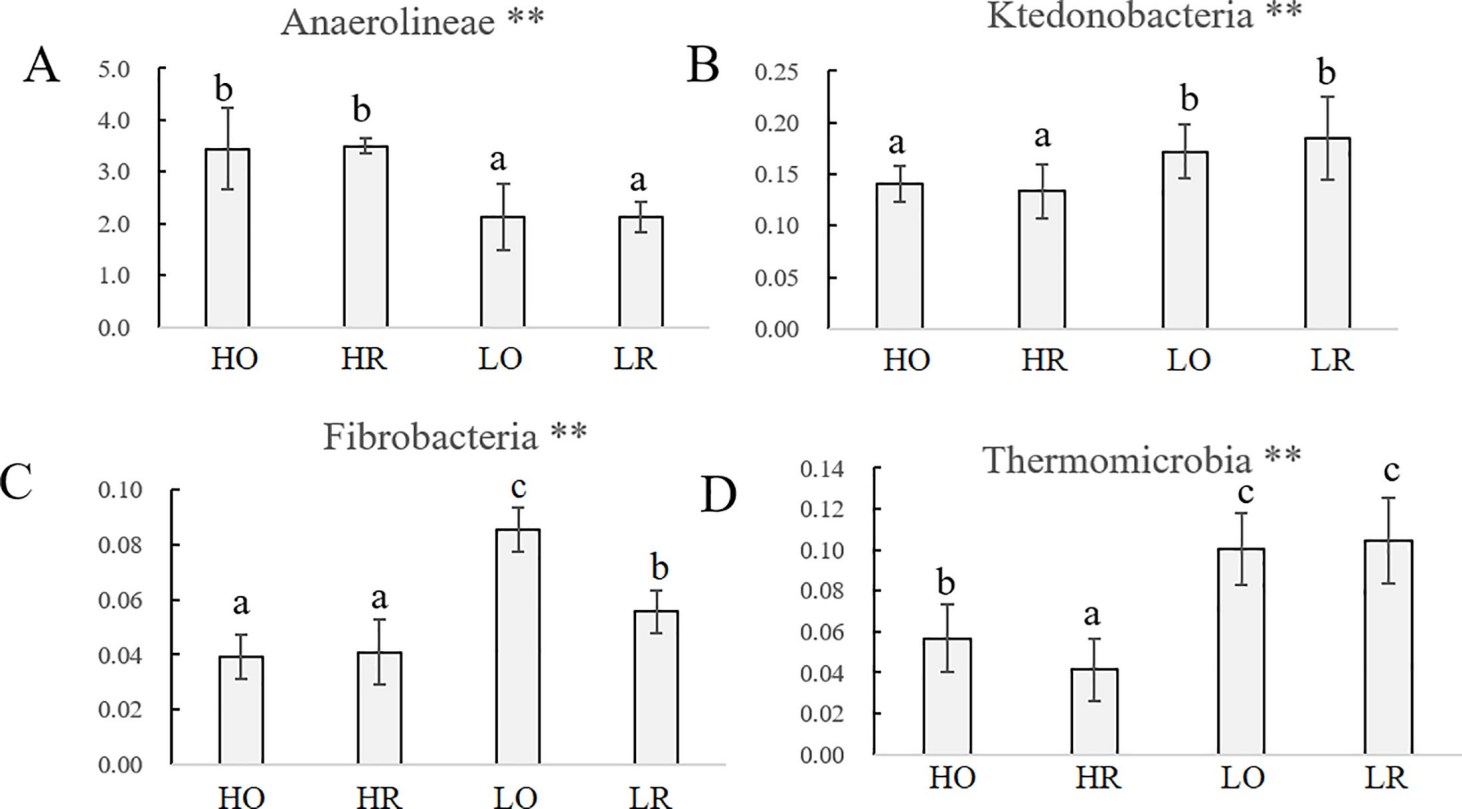
Average relative abundances of classes affected by fertilization regimes. The error bars indicate the standard deviations of the relative abundances between five replicate samples.

We also observed obvious difference in four classes of bacteria and archaea between low and high NPK soils, as shown in Fig 4. The proportion of the class Anaerolineae was higher under the high-NPK treatment than under the low-NPK treatment (Fig 4A, Table 4). In contrast, the percentage of the classes Ktedonobacteria, Fibrobacteria and Thermomicrobia were significantly (*P* < 0.05) higher in the low-NPK soils than in the high-NPK soils (Fig 4B–4D, respectively and Table 4).

**Fig 5:**
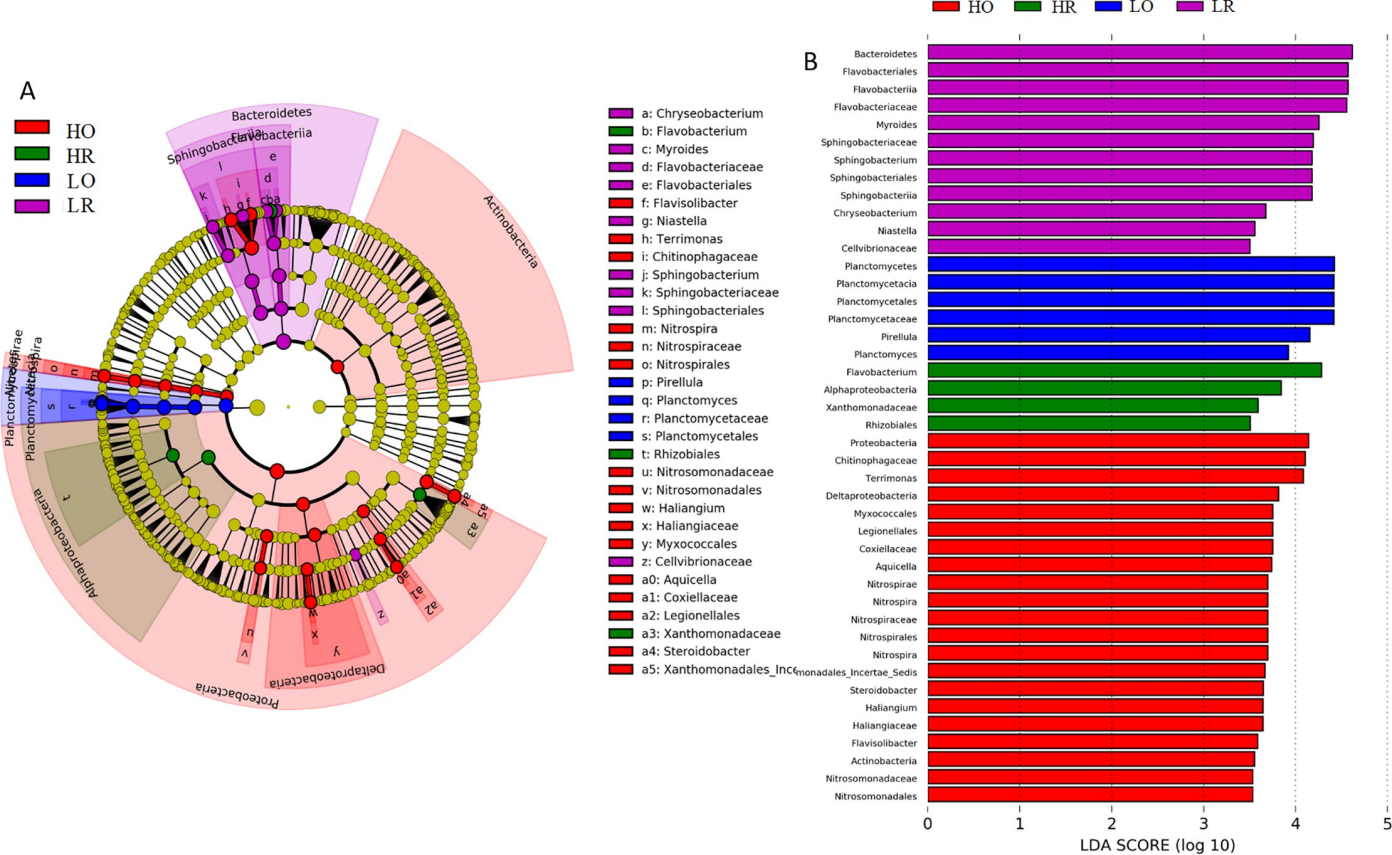
Distribution and representation of the taxa contributing to the different microbial communities in four soil samples. **A.** LEfSe taxonomic cladograms. Significantly discriminant taxon nodes are coloured. Branches are shaded according to the highest ranked group for the corresponding taxon. The yellow nodes represent the taxa with no significant differences among the sampled habitats. **B.** Histogram of LDA scores showing the discriminant taxa among four soil samples.

Furthermore, LEfSe analyses with an LDA threshold of 3.5 identified 43 bacterial taxa exhibiting significant differences among all the soil samples (Fig 5). Different number of taxa were enriched in the different soils (21 taxa in HO, 4 in HR, 6 in LO, 12 in HR) (Fig 5B). These groups primarily belonged to five phyla: Proteobacteria, Planctomycetes, Nitrospirae, Bacteroidetes and Actinobacteria (Fig 5A). In particular, bacterial taxa including the class Alphaproteobacteria, order Rhizobiales, family Xanthomonadaceae and genus *Flavobacterium* (S2A–S2D Fig, respectively) were identified as biomarkers in the HR soil samples.

### NMDS and RDA

**Fig 6:**
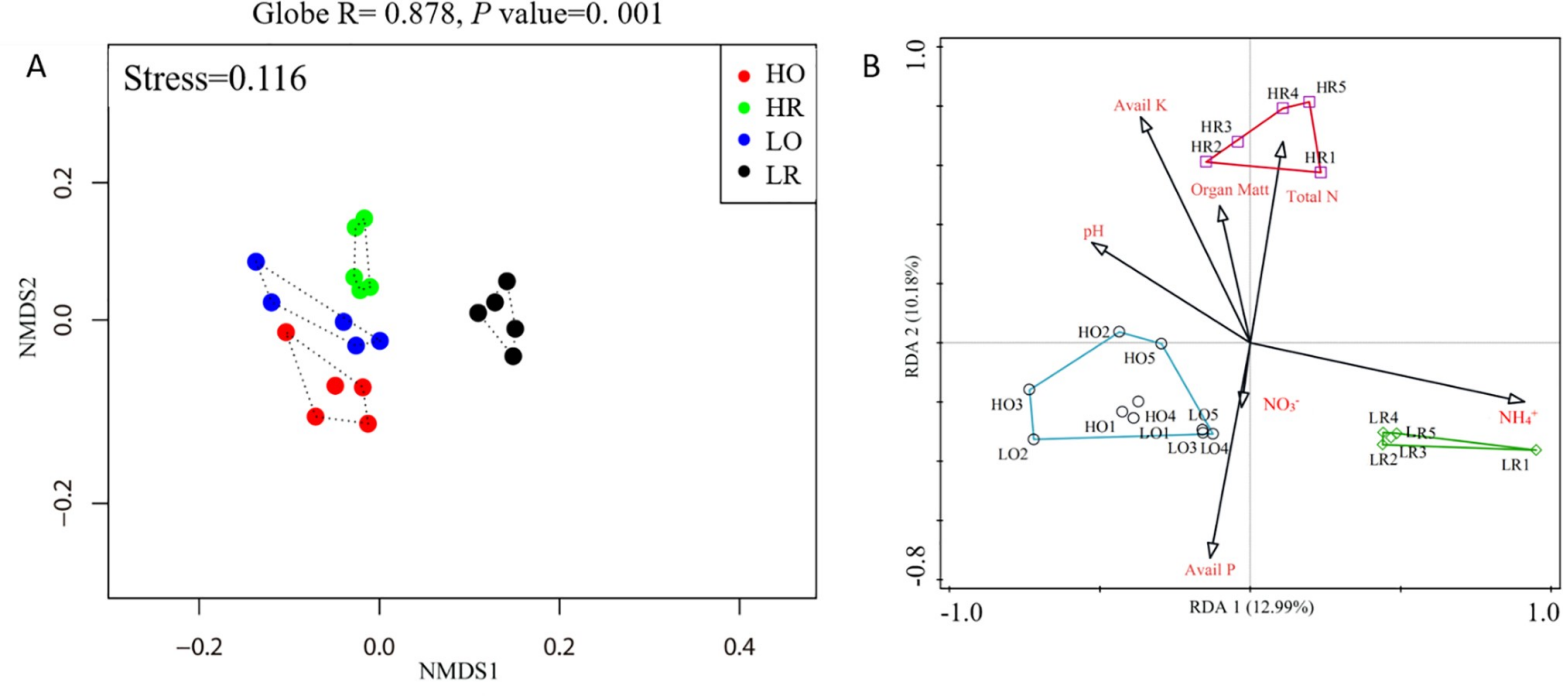
Nonmetric Multidimensional Scaling (NMDS) and Redundancy Analysis (RDA).

A graphic representation of the NMDS results allowed comparison of the samples based on weighted phylogenetic criteria and sequence alignment (Fig 6A). The LR samples were strongly separated from the three other soil samples along axis 1, and the HR samples were separated from the other samples along axis 2. The ANOSIM results showed that the bacterial communities in the four groups were significantly different and that the differences between groups were significantly greater than the differences within each group (Global R = 0.878, *P* = 0.001) (Fig 6A). The pairwise PERMANOVA comparisons were significantly different (*P* < 0.05, Table 5). We observed significant difference (*P* < 0.05) between HO and LR (R = 0.615) and between HR and LO (R = 0.367), which showed that the strength of the soil compartment on bacterial and archaeal recruitment differed among fertilization regimes. Greater differences were observed between the bulk and rhizosphere communities under the low-NPK treatment (LO/LR, R = 0.607) compared with the high-NPK treatment (HO/HR, R = 0.413) (Table 5). We also found a greater difference between the high- and low-NPK communities in the rhizosphere samples (HR/LR, R = 0.594) than in the bulk samples (HO/LO, R = 0.340) (Table 5).

**Table 5:** PERMANOVA pairwise comparisons of bacterial community composition.

Group	R statistic	Significance
HO/HR	0.4127	0.008
HO/LO	0.340393	0.017
HO/LR	0.614702	0.018
HR/LO	0.367154	0.011
HR/LR	0.593952	0.006
LO/LR	0.607256	0.01

The RDA results shown in Fig 6B revealed that axis 1 explained 12.99% of the total variance separating the rhizosphere (LR and HR) from the bulk (LO and HO) samples. Along axis 2, which explained 10.8% of the total variance, HR was strongly separated from the other three samples. The three most important factors affecting the changes in bacterial and archaeal communities were the soil concentrations of NH_4_^+^ (*F* = 2.1, *P* = 0.002), Avail K (*F* = 1.6, *P* = 0.012) and NO_3_^-^ (*F* = 1.5, *P* = 0.022), explaining 11.7%, 8.5% and 7.9% of the observed variance, respectively (S3 Table). All environmental variables together accounted for 50.8% of the microbial community changes between samples.

## Discussion

### Positive fertilizer and rhizosphere effects on bacterial and archaeal abundance

The relatively higher bacterial and archaeal abundance in high NPK soils (Fig 1) may be due to the higher content of nutrients required for bacterial and archaeal growth [35] in the high-NPK fertilizer treatments. Among these nutrients, the correlation analysis also showed that the concentrations of Avail K, total N and organic matter were directly proportional to the bacterial and archaeal abundances (S1 Table). These results may be related to the dominant Proteobacteria, which are heterotrophic and were the major microbiota component obtained from root exudates [36]. Alphaproteobacteria, Betaproteobacteria and Gammaproteobacteria in Proteobacteria are often characterized as rapidly growing r-strategists that respond positively to low-molecular-weight substrates [37], which were more abundant in the garlic rhizosphere than in the bulk soils in this study. In addition, Thaumarchaeota was the dominant (13.2–15.2%, S2A Fig) archaeal taxon in 20 samples, and studies of Thaumarchaeota have shown that these species play an important role in the biogeochemical cycling of nitrogen and carbon [38].

Arnault’s [39] research showed that the degradation of garlic tissue (leaves, stems and roots) that occurs during harvesting or straw return releases sulphurous volatiles such as thiosulphinates and zwiebelanes. These volatiles are converted into disulphides that show biocidal activities against fungi, nematodes and arthropods. This effect may lead to an increase in the number of bacteria competing with the above organisms for nutrients, which may explain the positive rhiosphere effects on the bacterial and archaeal abundance (Fig 1) found in this study. Moreover, the garlic rhizosphere exhibits a high content of soil organic carbon [40]. For example, dibutyl phthalate, which provides a carbon source for *Arthrobacter* sp. [41], may lead to the recruitment of specific soil microorganisms and affect root-knot nematode egg hatching [42], and thus increase the bacterial and archaeal abundance.

### Comparison of the bacterial and archaeal richness, evenness and diversity

The one way ANOVA result of Chao index indicated that high-NPK fertilization decreased the richness of bacteria. Although no soil properties were correlated with the Chao index, the total N was negatively and significantly correlated with ACE, which may be due to higher total N reducing the viability of certain microorganisms (Table 3). For example, some microorganisms in the classes Ktedonobacteria [43], Thermomicrobia [44] and Fibrobacteria [45] (Fig 4B, 4C and 4D, respectively) may show weakened growth and struggle to survive or even die when they do not adapt to high-nutrition conditions, resulting in a reduction in their abundance and a decrease in their richness in HO and HR.

In the high-NPK samples compared with the low-NPK samples, the richness decreased but the Shannon index increased (Table 3), possibly due to an increased evenness, which has been identified as a key factor in preserving the functional stability of an ecosystem [46].

### Bacterial and archaeal community composition

The examination of how the fertilizer doses and garlic roots shape the bacterial and archaeal community composition revealed differences between the high/low-NPK and bulk/rhizosphere samples at different classification levels.

The species of class Alphaproteobacteria are organotrophic, can thrive in nutrient-rich soils and utilize reduced forms of inorganic N (ammonia, nitrite or nitrate) as energy sources [47]. In this context, the high garlic yield in the high-NPK fertilizer treatments from 2011–2016 (S1 Fig) may benefit from the enrichment of Alphaproteobacteria in HR (Fig 5B and S2A Fig) due to their contribution to accelerating the N cycle [48]. Rhizobia naturally infect legumes as host plants, whereas some Rhizobium strains in the order Rhizobiales can form symbiotic relationships with nonlegume species such as Parasponia [49], radishes [50], Arabidopsis [51] and rice [52]. The enrichment of the order Rhizobiales in HR (Fig 5B and S2B Fig) and the higher proportion of the genus Rhizobium in HR and LR (S2C Fig) may indicate that garlic has the potential to form a symbiotic relationship with Rhizobia and thereby promote garlic growth.

We observed positive rhizosphere effects on the class Gammaproteobacteria (Fig 3D), and the family Xanthomonadaceae (S2C Fig) belonging to this class was dominant in HR. Xanthomonadaceae is a widespread family of bacteria in soil, including the plant-pathogenic genera *Xanthomonas*, *Xylella* and *Stenotrophomonas* [53], isolates that are recognized as important plant pathogens [54]. Fortunately, *Xanthomonas* and *Xylella* did not appear in all soil samples, but we found a higher *Stenotrophomonas* abundance in HR than in LR, LO and LR (Fig 2C). This finding indicates the potential disease danger (i.e. leaf scorch disease) [55] in garlic with the long-term application of large amounts of inorganic NPK.

The class Deltaproteobacteria was more abundant in bulk soil compared to the garlic rhizosphere, as also found in soybeans [56]. These changes in the percentage of specific taxa might result from changes in the composition of garlic root exudates during growth and development because garlic roots exhibit a reciprocal relationship with adjacent bacterial and archaeal groups in the rhizosphere [57]. Doolotkelvieva et al. [58] found that the class Flavobacteriia dominated the soybean rhizosphere, as also found in the garlic rhizosphere (Fig 3A). Among Flavobacteriia, several species of the genus *Flavobacterium* are associated with plant protection and growth promotion [59] and with bioremediation in soils [60] and marine sediments [61]. The LEfSe analysis revealed that *Flavobacterium* was the dominant group in HR (Fig 5B), which indicates the potential of these species in promoting the growth and increasing the garlic yield from 2011–2016 (S1 Fig). This result may guide the isolation and development of microbial agents such as plant growth-promoting rhizobacteria.

The classes Ardenticatenia and Caldilineae were more abundant in bulk soil (Fig 3C and 3D and Table 4). A previous study also indicated that microorganisms in these two classes could use oxygen as an electron acceptor and that the aerobic absorption of the matrix is a common feature [62]. The percentage of Thermomicrobia, many of which are thermophilic and have a broad chemoorganotrophic substrate specificity [63], decreased in the high-NPK group compared with the low-NPK group (Fig 4D and Table 4), indicating the negative influence of a high concentration on these groups. This finding was consistent with our study of the effects of 34 years of nitrogen fertilization on intensive black soil bacterial and archaeal communities in northeast China [6]. In contrast, Anaerolineae was dominant in the high-NPK samples compared with the low-NPK samples (Fig 4A). This result is consistent with the results of Wang et al. [64], who indicated that high NPK significantly stimulates the growth and production of Anaerolineae and that its abundance presents a positive relationship with the Avail P content. However, no significant difference in Avail P was found between the high- and low-NPK treatments (Table 2) in this study.

### Factors shaping the bacterial and archaeal community structure

The results from the NMDS analysis (Fig 6A) were consistent with the RDA results (Fig 6B), which indicated that bacterial communities in bulk soil (HO and LO) was more similar and significantly separated from those in the rhizosphere (HR and LR). The effect of soil compartment on the bacterial community structure were stronger than those of fertilization regime, indicating that microbial species respond more strongly to root exudates, as reported by Wang et al. [7]. The soil compartment effect on bacteria under the continuous cropping of garlic may be related to the root exudates. The accumulation of carbohydrates and amino acids in root exudates provides the required energy for certain species and promotes the growth and reproduction of rhizosphere bacteria [13]. In contrast, phenolic acids secreted by garlic roots inhibit the growth of beneficial soil microorganisms and exert their own toxic effects, disrupting the balance of the original microbial community and its structure in the rhizosphere of garlic [13].

However, the bacterial communities differed between HR and LR, which showed that fertilizer dose also significantly affected the bacterial communities, and many researchers agree on this point [6, 7, 65, 66]. The concentration of NH_4_^+^ in soil (contribution of 23%, *P* = 0.002) was the most important factor driving the changes in the bacterial community. This is consistent with the findings of Wang et al. [67], who concluded that NH_4_^+^ is the key factor affecting the population size of bacterial ammonia oxidizers. Our previous research [6] showed that the composition of the bacterial community was closely correlated with the NO_3_^−^ concentration in cultivated black soil, and we obtained the same result in this study. Similar to the results of the current study, Li et al. [68] found that the Avail K content was significantly associated with microbial changes in the rhizosphere under maize–peanut intercropping.

## Conclusion

This study systematically analysed the interactive effects of two important factors in a continuous cropping garlic system (fertilizer dose and soil compartment) on soil microbial communities. We found that the fertilizer dose impacted the bacterial and archaeal communities in rhizosphere differently than those in bulk soil, and these findings could be used to guide research priorities and management decisions. The changes in the soil concentrations of NH_4_^+^, Avail K and NO_3_^−^ appeared to change the community composition. The long-term use of high NPK fertilizer reduced the diversity and richness of bacteria. Significant differences in bacterial and archaeal community structure were found between the rhizosphere and bulk soils under different fertilization doses. Our results provide evidence that the influences of fertilizer dose and the soil compartment interact to shape the bacterial and archaeal communities in a continuous garlic cropping system, and we highlight the need to reframe the fertilizer and rhizosphere interaction as a dynamic process. However, whether the rules identified in this study are applicable to other continuous cropping systems and which changes in microbial functions correspond to changes in these microbial indicators affecting the yield and garlic health are issues to be further explored in the future.

## Supporting information

S1 Fig Garlic yield in the high (H) and low (L) NPK fertilizer treatments from 2011 to 2016. (TIF)

S2 Fig Differently enriched taxa in soil groups based on abundance within phylogenetic lineages (LEfSe analysis). (TIF)

S1 Table Pearson’s correlation coefficients between soil chemical properties and number of 16S rRNA gene copies. (DOCX)

S2 Table Pearson’s corrections between soil properties and ɑ-diversity. (DOCX)

S3 Table Forward selection results of RDA analysis. (DOCX)

S1 File (XLSX)

S2 File (XLSX)

S3 File (XLSX)

S4 File (XLSX)
