# Supplementary figures and images for: Comparison of bacterial and archaeal communities in two fertilizer doses and soil compartments under continuous cultivation system of garlic
Source: PLoS One. 2021 May 14;16(5):e0250571. doi: 10.1371/journal.pone.0250571 (PMC8121308; doi:10.1371/journal.pone.0250571)

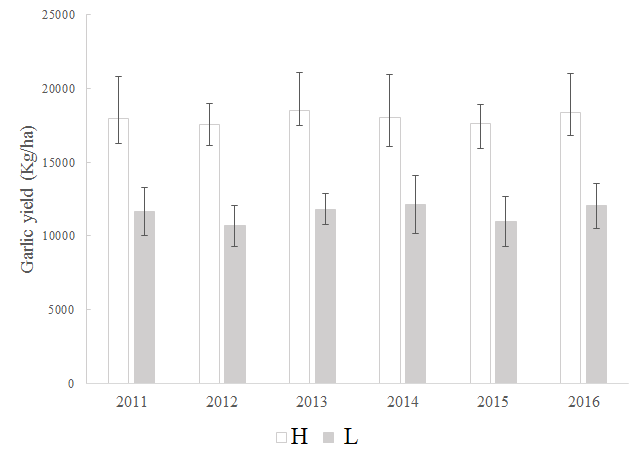

Supplement: S1 Fig — (TIF) [file pone.0250571.s001.tif]

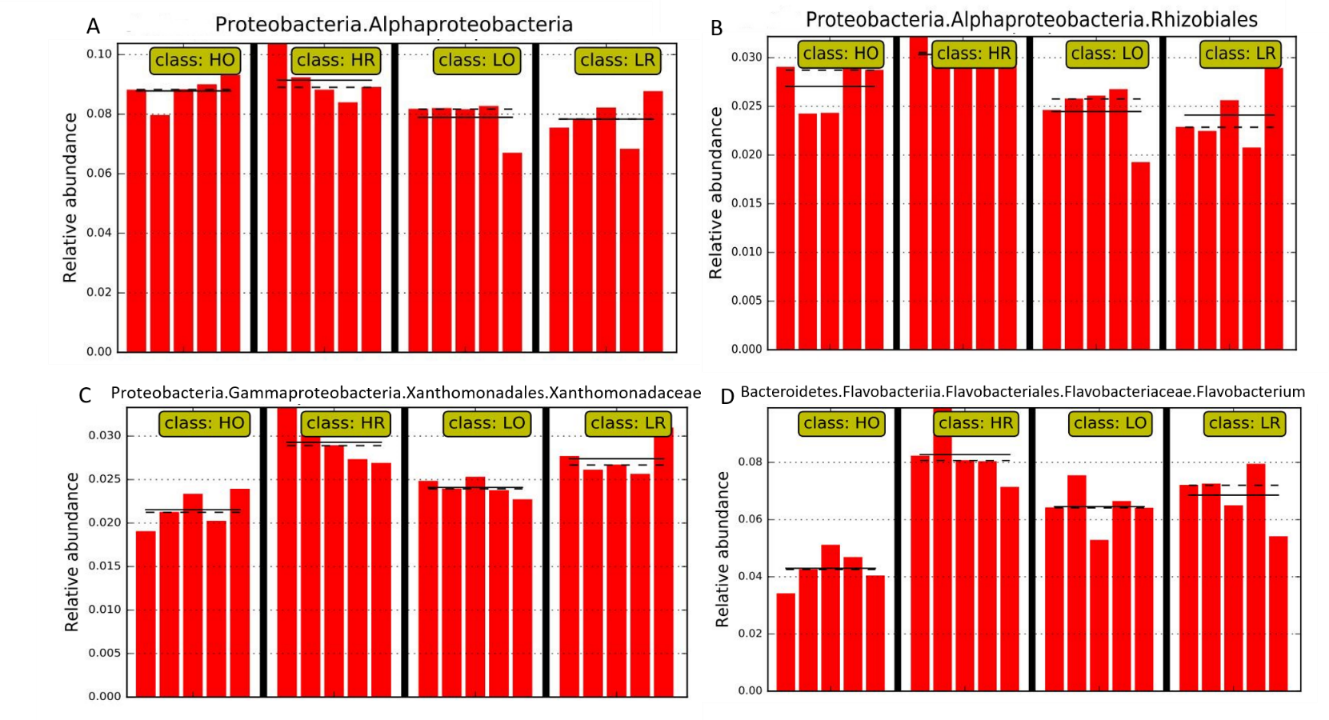

Supplement: S2 Fig — (TIF) [file pone.0250571.s002.tif]
