# Supplementary material for: Comparison of bacterial and archaeal communities in two fertilizer doses and soil compartments under continuous cultivation system of garlic
Source: PLoS One. 2021 May 14;16(5):e0250571. doi: 10.1371/journal.pone.0250571 (PMC8121308; doi:10.1371/journal.pone.0250571)
Supplement: S1 Table — (DOCX) [file pone.0250571.s003.docx]

**S1 Table Pearson's correlation coefficients between soil chemical properties and number of 16S rRNA gene copies.**

|  | pH | Avail K | Total N | Avail P | NH_4_^+^ -N | NO_3_^-^-N | Organic matter |
| --- | --- | --- | --- | --- | --- | --- | --- |
| F-value | 0.215 | 0.895^**^ | 0.955^**^ | -0.491 | -0.210 | 0.225 | 0.664^*^ |
| P-value | 0.502 | 0.000 | 0.000 | 0.105 | 0.512 | 0.484 | 0.224 |

**P* < 0.05, ***P* < 0.01
