# Supplementary material for: Comparison of bacterial and archaeal communities in two fertilizer doses and soil compartments under continuous cultivation system of garlic
Source: PLoS One. 2021 May 14;16(5):e0250571. doi: 10.1371/journal.pone.0250571 (PMC8121308; doi:10.1371/journal.pone.0250571)
Supplement: S2 Table — (DOCX) [file pone.0250571.s004.docx]

**S2 Table Pearson’s corrections between soil properties and ɑ-diversity**

|  | pH | Avail K | Total N | Avail P | NH_4_^+^ | NO_3_^–^ | Organic matter |
| --- | --- | --- | --- | --- | --- | --- | --- |
| Ace | 0.168 | -0.155 | -0.447^*^ | 0.382 | -0.317 | -0.208 | -0.176 |
| Invsimpson | -0.507^*^ | -0.223 | 0.128 | -0.046 | 0.045 | -0.153 | -0.296 |
| Shannon | -0.407 | -0.235 | 0.115 | -0.105 | 0.469* | -0.170 | -0.203 |
| Chao | 0.094 | -0.271 | -0.442 | 0.321 | -0.331 | -0.325 | -0.234 |

**P* < 0.05
