# Supplementary material for: Comparison of bacterial and archaeal communities in two fertilizer doses and soil compartments under continuous cultivation system of garlic
Source: PLoS One. 2021 May 14;16(5):e0250571. doi: 10.1371/journal.pone.0250571 (PMC8121308; doi:10.1371/journal.pone.0250571)
Supplement: S3 Table — (DOCX) [file pone.0250571.s005.docx]

**S3 Table Forward selection results of RDA analysis**

| Name | Explains % | pseudo-F | P-value |
| --- | --- | --- | --- |
| NH_4_^+^ -N | 11.7 | 2.1 | 0.002 |
| Avail K | 8.5 | 1.6 | 0.012 |
| NO_3_^-^ -N | 7.9 | 1.5 | 0.022 |
| Total N | 6.6 | 1.3 | 0.146 |
| Avail P | 6.2 | 1.3 | 0.146 |
| Organic matter | 5.1 | 1 | 0.446 |
| pH | 4.8 | 1 | 0.49 |
